# 1-(1,3-Benzothia­zol-2-yl)-3-(4-chloro­benzo­yl)thio­urea

**DOI:** 10.1107/S160053680903743X

**Published:** 2009-09-26

**Authors:** M. Sukeri M. Yusof, Zakaria S. Aishah, Wan M. Khairul, Bohari M. Yamin

**Affiliations:** a Department of Chemical Sciences, Faculty of Science and Technology, Universiti Malaysia Terengganu, Mengabang Telipot, 21030 Kuala Terengganu, Malaysia; b School of Chemical Sciences and Food Technology, Universiti Kebangsaan Malaysia, 43600 Bangi, Selangor, Malaysia

## Abstract

The title compound, C_15_H_10_ClN_3_OS_2_, adopts a *cis–trans* configuration across the thio­urea C—N bonds with respect to the positions of the benzothia­zole and 4-chloro­benzoyl groups relative to thiono S atom. An intra­molecular N—H⋯O hydrogen bond is present. In the crystal structure, mol­ecules are linked by a weak inter­molecular N—H⋯S hydrogen bond, forming centrosymmetric dimers.

## Related literature

For the biological activity of thia­diazo­les, see: Shukla & Srivastava (2008 ▶); Göblyös *et al.* (2005 ▶); Terzioglu & Gürsoy (2003 ▶); Rana *et al.* (2008 ▶). For their potential as insecticides and fungicides, see: Jian *et al.* (2005 ▶). For C—S and C—O bond lengths, see: Saeed & Flörke (2006 ▶); Yamin & Yusof (2003 ▶). For the structures of other benzoyl­thio­urea derivatives, see: Dillen *et al.* (2006 ▶); Khawar Rauf *et al.* (2006 ▶); Weiqun *et al.* (2004 ▶).
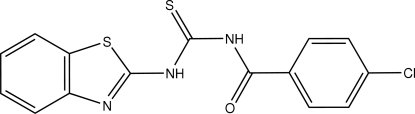


## Experimental

### 

#### Crystal data

C_15_H_10_ClN_3_OS_2_
*M* *_r_* = 347.83
Monoclinic, 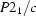

*a* = 11.726 (2) Å
*b* = 17.934 (4) Å
*c* = 7.2617 (16) Å
β = 96.848 (4)°
*V* = 1516.1 (6) Å^3^
*Z* = 4
Mo *K*α radiation
μ = 0.53 mm^−1^
*T* = 298 K
0.55 × 0.42 × 0.40 mm

#### Data collection

Bruker SMART APEX CCD area-detector diffractometer
Absorption correction: multi-scan (*SADABS*; Bruker, 2000 ▶) *T*_min_ = 0.759, *T*_max_ = 0.816
11030 measured reflections
3772 independent reflections
2891 reflections with *I* > 2σ(*I*)
*R*_int_ = 0.022

#### Refinement

*R*[*F* ^2^ > 2σ(*F* ^2^)] = 0.039
*wR*(*F* ^2^) = 0.109
*S* = 1.05
3772 reflections
199 parameters
H-atom parameters constrained
Δρ_max_ = 0.30 e Å^−3^
Δρ_min_ = −0.22 e Å^−3^

### 

Data collection: *SMART* (Bruker, 2000 ▶); cell refinement: *SAINT* (Bruker, 2000 ▶); data reduction: *SAINT*; program(s) used to solve structure: *SHELXS97* (Sheldrick, 2008 ▶); program(s) used to refine structure: *SHELXL97* (Sheldrick, 2008 ▶); molecular graphics: *SHELXTL* (Sheldrick, 2008 ▶); software used to prepare material for publication: *SHELXTL*, *PARST* (Nardelli, 1995 ▶) and *PLATON* (Spek, 2009 ▶).

## Supplementary Material

Crystal structure: contains datablocks global, I. DOI: 10.1107/S160053680903743X/vm2003sup1.cif

Structure factors: contains datablocks I. DOI: 10.1107/S160053680903743X/vm2003Isup2.hkl

Additional supplementary materials:  crystallographic information; 3D view; checkCIF report

## Figures and Tables

**Table 1:** Hydrogen-bond geometry (Å, °)

*D*—H⋯*A*	*D*—H	H⋯*A*	*D*⋯*A*	*D*—H⋯*A*
N2—H2*A*⋯O1	0.86	1.88	2.6056 (19)	141
N1—H1*A*⋯S1^i^	0.86	2.75	3.5377 (17)	152

Symmetry code: (i) 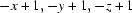.

**Fig. 1.**
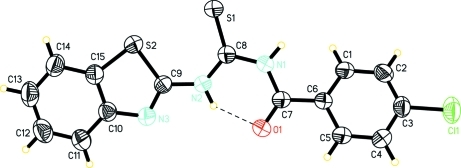
The molecule of (I) showing the atom-labelling scheme. Displacement ellipsoids are drawn at the 50% probability level. Dashed lines indicate hydrogen bonds.

**Fig. 2.**
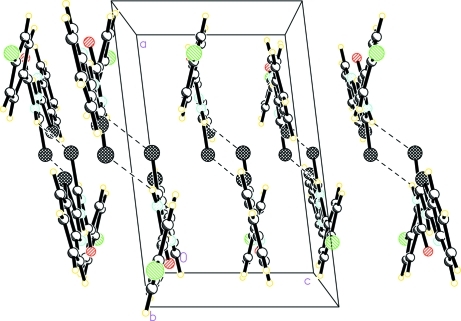
Packing diagram of compound (I), viewed down the b axis. The dashed lines denote the N—H···S hydrogen bond.

## supplementary crystallographic information

### Comment

For the past few decades, heterocycles featuring thiadiazoles which consist of sulfur and nitrogen have been developed consistenly to act as drugs as well as to play an active role in numerous biological activities (Shukla & Srivastava 2008; Göblyös *et al.* 2005). These derivatives have been shown to exhibit anticancer, antitubercular and anticonvulsant activities (Terzioglu & Gürsoy 2003; Rana *et al.* 2008). In agriculture, thiadiazole derivatives have a potential as insecticide and fungicide (Jian *et al.* 2005). The title compound (I), adopts *cis-trans* configuration with respect to the positions of the benzothiazole and 4-chlorobenzoyl groups relative to the thiono S atom, across their C—N bonds (Fig 1). The central carbonyl thiourea moiety (S1/C8/N1/N2/C7/O1), phenyl ring (C1—C6) and benzothiazole (S2/N3/C9—C15) groups are all planar, with a maximum deviation of 0.021 (1)Å for atom C10 from the least-squares plane. The central carbonyl thiourea fragment makes dihedral angles of 24.09 (7)° and 4.58 (4)° with the phenyl ring and benzothiazole group, respectively. The two aryl rings are inclined to each other at an angle of 28.42 (8)°. The C8—S1 and C7—O1 bond length show the expected double bond character of 1.6570 (17)Å and 1.2245 (19)Å (Saeed & Flörke 2006; Yamin & Yusof 2003). The N1—C8 is longer than N2—C8 by 0.045 Å, similar to other benzoylthiourea derivatives (Dillen *et al.* 2006; Khawar Rauf *et al.* 2006) which is probably due to the intramolecular hydrogen bonding interaction (Weiqun *et al.* 2004).

There is an intramolecular hydrogen bond, N2—H2···O1 forming a pseudo-six-membered ring, O1···H2—N2—C8—N1—C7—O1 (Fig.1). In the crystal structure, the molecules are linked by a weak intermolecular interaction N1—H1A···S1 (symmetry codes as in Table 1) forming dimers (Fig. 2).

### Experimental

To a stirring acetone solution (75 ml) of 4-chlorobenzoyl chloride (2.0 g, 11.4 mmol) and ammoniumthiocyanate (0.87 g, 11.4 mmol), 2-aminobenzothiazole (1.17 g, 11.4 mmol) in 40 ml of acetone was added dropwise. The solution mixture was put at reflux for 1 h. The resulting solution was poured into a beaker containing some ice blocks. The light yellow precipitate was filtered off and washed with distilled water and cold ethanol before dried under vacuum. Good quality crystals were obtained by recrystallization from DMSO.

### Refinement

After their location in the difference map, all H-atoms were fixed geometrically at ideal positions and allowed to ride on the parent C or N atoms with C—H = 0.93Å and N—H = 0.86Å with *U*_iso_(H)= 1.2 (CH and NH).

### Crystal data

**Table:**

C_15_H_10_ClN_3_OS_2_	*F*(000) = 712
*M**_r_* = 347.83	*D*_x_ = 1.524 Mg m^−^^3^
Monoclinic, *P*2_1_/*c*	Mo *K*α radiation, λ = 0.71073 Å
Hall symbol: -P 2ybc	Cell parameters from 906 reflections
*a* = 11.726 (2) Å	θ = 1.8–28.3°
*b* = 17.934 (4) Å	µ = 0.53 mm^−^^1^
*c* = 7.2617 (16) Å	*T* = 298 K
β = 96.848 (4)°	Block, light yellow
*V* = 1516.1 (6) Å^3^	0.55 × 0.42 × 0.40 mm
*Z* = 4	

### Data collection

**Table:**

Bruker SMART APEX CCD area-detector diffractometer	3772 independent reflections
Radiation source: fine-focus sealed tube	2891 reflections with *I* > 2σ(*I*)
graphite	*R*_int_ = 0.022
Detector resolution: 83.66 pixels mm^-1^	θ_max_ = 28.3°, θ_min_ = 1.8°
ω scans	*h* = −15→15
Absorption correction: multi-scan (*SADABS*; Bruker, 2000)	*k* = −23→20
*T*_min_ = 0.759, *T*_max_ = 0.816	*l* = −8→9
11030 measured reflections	

### Refinement

**Table:**

Refinement on *F*^2^	Primary atom site location: structure-invariant direct methods
Least-squares matrix: full	Secondary atom site location: difference Fourier map
*R*[*F*^2^ > 2σ(*F*^2^)] = 0.039	Hydrogen site location: inferred from neighbouring sites
*wR*(*F*^2^) = 0.109	H-atom parameters constrained
*S* = 1.05	*w* = 1/[σ^2^(*F*_o_^2^) + (0.0606*P*)^2^ + 0.1737*P*] where *P* = (*F*_o_^2^ + 2*F*_c_^2^)/3
3772 reflections	(Δ/σ)_max_ < 0.001
199 parameters	Δρ_max_ = 0.30 e Å^−^^3^
0 restraints	Δρ_min_ = −0.22 e Å^−^^3^

### Special details

**Table:**

Geometry. All e.s.d.'s (except the e.s.d. in the dihedral angle between two l.s. planes) are estimated using the full covariance matrix. The cell e.s.d.'s are taken into account individually in the estimation of e.s.d.'s in distances, angles and torsion angles; correlations between e.s.d.'s in cell parameters are only used when they are defined by crystal symmetry. An approximate (isotropic) treatment of cell e.s.d.'s is used for estimating e.s.d.'s involving l.s. planes.
Refinement. Refinement of *F*^2^ against ALL reflections. The weighted *R*-factor *wR* and goodness of fit *S* are based on *F*^2^, conventional *R*-factors *R* are based on *F*, with *F* set to zero for negative *F*^2^. The threshold expression of *F*^2^ > σ(*F*^2^) is used only for calculating *R*-factors(gt) *etc*. and is not relevant to the choice of reflections for refinement. *R*-factors based on *F*^2^ are statistically about twice as large as those based on *F*, and *R*- factors based on ALL data will be even larger.

### Fractional atomic coordinates and isotropic or equivalent isotropic displacement parameters (Å^2^)

**Table:**

	*x*	*y*	*z*	*U*_iso_*/*U*_eq_	
Cl1	0.84415 (5)	0.83795 (3)	0.39677 (9)	0.07175 (19)	
S1	0.49665 (4)	0.38829 (3)	0.39437 (8)	0.05844 (16)	
S2	0.58802 (4)	0.22891 (2)	0.41252 (6)	0.04563 (13)	
O1	0.86117 (10)	0.46684 (7)	0.3645 (2)	0.0549 (3)	
N3	0.80322 (12)	0.24282 (8)	0.3637 (2)	0.0474 (3)	
N1	0.67029 (11)	0.48112 (8)	0.3894 (2)	0.0450 (3)	
H1A	0.6183	0.5141	0.4005	0.054*	
N2	0.71709 (11)	0.35696 (7)	0.3786 (2)	0.0434 (3)	
H2A	0.7842	0.3750	0.3695	0.052*	
C1	0.70106 (14)	0.63867 (10)	0.3181 (3)	0.0491 (4)	
H1	0.6291	0.6194	0.2754	0.059*	
C2	0.71738 (15)	0.71472 (10)	0.3236 (3)	0.0520 (4)	
H2	0.6570	0.7469	0.2855	0.062*	
C3	0.82429 (16)	0.74219 (10)	0.3862 (3)	0.0489 (4)	
C4	0.91544 (15)	0.69616 (11)	0.4420 (3)	0.0551 (5)	
H4	0.9873	0.7159	0.4832	0.066*	
C5	0.89835 (14)	0.61965 (10)	0.4358 (3)	0.0507 (4)	
H5	0.9594	0.5877	0.4722	0.061*	
C6	0.79104 (13)	0.59043 (9)	0.3759 (2)	0.0418 (4)	
C7	0.77928 (13)	0.50832 (10)	0.3753 (2)	0.0420 (4)	
C8	0.63429 (13)	0.40769 (9)	0.3881 (2)	0.0417 (4)	
C9	0.71104 (14)	0.27973 (9)	0.3813 (2)	0.0404 (4)	
C10	0.78156 (15)	0.16707 (10)	0.3728 (2)	0.0459 (4)	
C11	0.86167 (18)	0.11049 (11)	0.3579 (3)	0.0612 (5)	
H11	0.9372	0.1220	0.3421	0.073*	
C12	0.82695 (19)	0.03736 (12)	0.3672 (3)	0.0685 (6)	
H12	0.8797	−0.0007	0.3568	0.082*	
C13	0.7148 (2)	0.01951 (11)	0.3915 (3)	0.0657 (6)	
H13	0.6932	−0.0303	0.3964	0.079*	
C14	0.63489 (17)	0.07448 (10)	0.4087 (3)	0.0574 (5)	
H14	0.5599	0.0624	0.4266	0.069*	
C15	0.66891 (15)	0.14852 (9)	0.3988 (2)	0.0447 (4)	

### Atomic displacement parameters (Å^2^)

**Table:**

	*U*^11^	*U*^22^	*U*^33^	*U*^12^	*U*^13^	*U*^23^
Cl1	0.0907 (4)	0.0384 (3)	0.0882 (4)	−0.0049 (2)	0.0194 (3)	−0.0016 (2)
S1	0.0365 (2)	0.0450 (3)	0.0947 (4)	−0.00093 (18)	0.0117 (2)	−0.0050 (2)
S2	0.0387 (2)	0.0442 (2)	0.0534 (3)	−0.00318 (16)	0.00325 (18)	0.00143 (18)
O1	0.0381 (6)	0.0427 (7)	0.0842 (10)	0.0047 (5)	0.0088 (6)	−0.0007 (6)
N3	0.0428 (7)	0.0402 (8)	0.0602 (10)	−0.0005 (6)	0.0099 (7)	−0.0024 (6)
N1	0.0356 (7)	0.0381 (7)	0.0618 (9)	0.0029 (5)	0.0076 (6)	−0.0010 (6)
N2	0.0352 (6)	0.0381 (7)	0.0567 (9)	−0.0015 (5)	0.0045 (6)	−0.0020 (6)
C1	0.0366 (8)	0.0454 (9)	0.0649 (12)	0.0015 (7)	0.0046 (8)	0.0055 (8)
C2	0.0449 (9)	0.0453 (10)	0.0664 (12)	0.0083 (7)	0.0085 (8)	0.0083 (8)
C3	0.0570 (10)	0.0360 (8)	0.0557 (11)	−0.0018 (7)	0.0150 (8)	0.0002 (7)
C4	0.0428 (9)	0.0491 (10)	0.0727 (13)	−0.0069 (8)	0.0042 (8)	−0.0027 (9)
C5	0.0361 (8)	0.0456 (10)	0.0696 (12)	0.0023 (7)	0.0037 (8)	0.0028 (8)
C6	0.0355 (7)	0.0401 (9)	0.0503 (10)	0.0012 (6)	0.0071 (7)	0.0008 (7)
C7	0.0353 (7)	0.0438 (9)	0.0469 (9)	0.0007 (6)	0.0043 (7)	0.0009 (7)
C8	0.0391 (8)	0.0404 (8)	0.0455 (9)	0.0001 (6)	0.0043 (7)	−0.0014 (7)
C9	0.0387 (8)	0.0399 (8)	0.0419 (9)	−0.0021 (6)	0.0018 (6)	−0.0021 (6)
C10	0.0486 (9)	0.0411 (9)	0.0476 (10)	0.0009 (7)	0.0034 (7)	−0.0031 (7)
C11	0.0588 (11)	0.0462 (10)	0.0798 (14)	0.0061 (8)	0.0135 (10)	−0.0040 (9)
C12	0.0754 (14)	0.0442 (11)	0.0844 (16)	0.0115 (10)	0.0034 (12)	−0.0080 (10)
C13	0.0804 (15)	0.0391 (10)	0.0735 (14)	−0.0046 (10)	−0.0076 (11)	0.0004 (9)
C14	0.0605 (11)	0.0462 (10)	0.0628 (12)	−0.0098 (9)	−0.0035 (9)	0.0046 (8)
C15	0.0482 (9)	0.0414 (9)	0.0427 (9)	−0.0020 (7)	−0.0018 (7)	0.0006 (7)

### Geometric parameters (Å, °)

**Table:**

Cl1—C3	1.7334 (19)	C2—H2	0.9300
S1—C8	1.6570 (17)	C3—C4	1.373 (3)
S2—C15	1.7352 (18)	C4—C5	1.386 (3)
S2—C9	1.7438 (17)	C4—H4	0.9300
O1—C7	1.2245 (19)	C5—C6	1.384 (2)
N3—C9	1.287 (2)	C5—H5	0.9300
N3—C10	1.385 (2)	C6—C7	1.479 (2)
N1—C8	1.383 (2)	C10—C15	1.397 (2)
N1—C7	1.383 (2)	C10—C11	1.396 (3)
N1—H1A	0.8600	C11—C12	1.377 (3)
N2—C8	1.338 (2)	C11—H11	0.9300
N2—C9	1.387 (2)	C12—C13	1.385 (3)
N2—H2A	0.8600	C12—H12	0.9300
C1—C2	1.377 (2)	C13—C14	1.376 (3)
C1—C6	1.390 (2)	C13—H13	0.9300
C1—H1	0.9300	C14—C15	1.391 (2)
C2—C3	1.373 (3)	C14—H14	0.9300
			
C15—S2—C9	87.73 (8)	O1—C7—C6	122.10 (15)
C9—N3—C10	109.80 (14)	N1—C7—C6	115.95 (14)
C8—N1—C7	128.28 (14)	N2—C8—N1	115.16 (14)
C8—N1—H1A	115.9	N2—C8—S1	125.01 (13)
C7—N1—H1A	115.9	N1—C8—S1	119.83 (12)
C8—N2—C9	129.75 (14)	N3—C9—N2	117.86 (15)
C8—N2—H2A	115.1	N3—C9—S2	117.51 (13)
C9—N2—H2A	115.1	N2—C9—S2	124.62 (12)
C2—C1—C6	120.65 (16)	N3—C10—C15	114.96 (15)
C2—C1—H1	119.7	N3—C10—C11	125.47 (17)
C6—C1—H1	119.7	C15—C10—C11	119.57 (17)
C3—C2—C1	118.89 (16)	C12—C11—C10	118.9 (2)
C3—C2—H2	120.6	C12—C11—H11	120.6
C1—C2—H2	120.6	C10—C11—H11	120.6
C4—C3—C2	122.00 (17)	C11—C12—C13	121.1 (2)
C4—C3—Cl1	119.14 (15)	C11—C12—H12	119.4
C2—C3—Cl1	118.86 (14)	C13—C12—H12	119.4
C3—C4—C5	118.75 (17)	C14—C13—C12	120.87 (19)
C3—C4—H4	120.6	C14—C13—H13	119.6
C5—C4—H4	120.6	C12—C13—H13	119.6
C6—C5—C4	120.48 (16)	C13—C14—C15	118.47 (19)
C6—C5—H5	119.8	C13—C14—H14	120.8
C4—C5—H5	119.8	C15—C14—H14	120.8
C5—C6—C1	119.21 (16)	C14—C15—C10	121.07 (17)
C5—C6—C7	117.30 (14)	C14—C15—S2	128.91 (15)
C1—C6—C7	123.48 (15)	C10—C15—S2	110.01 (13)
O1—C7—N1	121.94 (16)		
			
C6—C1—C2—C3	0.3 (3)	C10—N3—C9—S2	0.4 (2)
C1—C2—C3—C4	0.5 (3)	C8—N2—C9—N3	177.61 (17)
C1—C2—C3—Cl1	−178.90 (15)	C8—N2—C9—S2	−4.0 (3)
C2—C3—C4—C5	−0.4 (3)	C15—S2—C9—N3	−0.44 (15)
Cl1—C3—C4—C5	179.00 (15)	C15—S2—C9—N2	−178.82 (15)
C3—C4—C5—C6	−0.5 (3)	C9—N3—C10—C15	−0.2 (2)
C4—C5—C6—C1	1.4 (3)	C9—N3—C10—C11	179.64 (19)
C4—C5—C6—C7	−179.30 (17)	N3—C10—C11—C12	−179.08 (18)
C2—C1—C6—C5	−1.3 (3)	C15—C10—C11—C12	0.8 (3)
C2—C1—C6—C7	179.44 (17)	C10—C11—C12—C13	−0.3 (3)
C8—N1—C7—O1	−2.2 (3)	C11—C12—C13—C14	−0.5 (3)
C8—N1—C7—C6	178.38 (16)	C12—C13—C14—C15	0.8 (3)
C5—C6—C7—O1	−24.7 (3)	C13—C14—C15—C10	−0.3 (3)
C1—C6—C7—O1	154.64 (18)	C13—C14—C15—S2	179.10 (15)
C5—C6—C7—N1	154.75 (16)	N3—C10—C15—C14	179.37 (16)
C1—C6—C7—N1	−25.9 (2)	C11—C10—C15—C14	−0.5 (3)
C9—N2—C8—N1	177.45 (16)	N3—C10—C15—S2	−0.1 (2)
C9—N2—C8—S1	−3.6 (3)	C11—C10—C15—S2	−179.97 (15)
C7—N1—C8—N2	2.7 (3)	C9—S2—C15—C14	−179.14 (18)
C7—N1—C8—S1	−176.34 (14)	C9—S2—C15—C10	0.28 (13)
C10—N3—C9—N2	178.94 (14)		

### Hydrogen-bond geometry (Å, °)

**Table:**

*D*—H···*A*	*D*—H	H···*A*	*D*···*A*	*D*—H···*A*
N2—H2A···O1	0.86	1.88	2.6056 (19)	141
N1—H1A···S1^i^	0.86	2.75	3.5377 (17)	152
C5—H5···O1^ii^	0.93	2.49	3.389 (2)	163

Symmetry codes:  (i) −*x*+1, −*y*+1, −*z*+1; (ii) −*x*+2, −*y*+1, −*z*+1.
